# Differential diagnosis of thickened myocardium: an illustrative MRI review

**DOI:** 10.1007/s13244-018-0655-9

**Published:** 2018-10-09

**Authors:** Cristina Méndez, Rafaela Soler, Esther Rodríguez, Roberto Barriales, Juan Pablo Ochoa, Lorenzo Monserrat

**Affiliations:** 1 Radiology Department, Complexo Hospitalario Universitario A Coruña, Instituto de Investigación Biomédica de A Coruña (INIBIC), Servizo Galego de Saúde (SERGAS), Universidade da Coruña, Xubias de Arriba 86, 15006 A Coruña, Spain; 2 Cardiology Department, Complexo Hospitalario Universitario A Coruña, Instituto de Investigación Biomédica de A Coruña (INIBIC), Servizo Galego de Saúde (SERGAS), Universidade da Coruña, Xubias de Arriba, 84, 15006 A Coruña, Spain

**Keywords:** Cardiac magnetic resonance, Hypertrophic cardiomyopathy, Myocardial thickening, Myocardial hypertrophy, Cardiomyopathies

## Abstract

**Objectives:**

The purpose of this article is to describe the key cardiac magnetic resonance imaging (MRI) features to differentiate hypertrophic cardiomyopathy (HCM) phenotypes from other causes of myocardial thickening that may mimic them.

**Conclusions:**

Many causes of myocardial thickening may mimic different HCM phenotypes. The unique ability of cardiac MRI to facilitate tissue characterisation may help to establish the aetiology of myocardial thickening, which is essential to differentiate it from HCM phenotypes and for appropriate management.

**Teaching points:**

• *Many causes of myocardial thickening may mimic different HCM phenotypes.*

• *Differential diagnosis between myocardial thickening aetiology and HCM phenotypes may be challenging.*

• *Cardiac MRI is essential to differentiate the aetiology of myocardial thickening from HCM phenotypes.*

## Introduction

Hypertrophic cardiomyopathy (HCM) is the most common genetic cardiovascular disorder worldwide, with a prevalence of 1 in 500 in the general population [1]. It is characterised by an unexplained left ventricular (LV) hypertrophy in the absence of other disease entities that may lead to inappropriate myocardial wall thickening caused by pressure/volume overload, infiltrative disorders, athlete’s heart or neoplastic infiltration [2–5]. For HCM diagnosis, international guidelines advocate using a wall thickness cut-off of 15 mm in one or more myocardial segments, measured by any imaging technique [6, 7].

Echocardiography is the most commonly used imaging modality in the evaluation of HCM. When the HCM phenotype is fully expressed, echocardiography generally allows a reliable and unequivocal diagnosis. Occasionally, however, the differential diagnosis among the broad range of phenotypic expressions of HCM and other causes of myocardial thickening may be challenging. Tissue characterisation, which is limited with echocardiography, could provide additional diagnostic information [5].

Cardiac magnetic resonance imaging (MRI) has evolved into a multiparametric imaging modality allowing a truly comprehensive picture of HCM, providing information regarding various phenotypes, their functional and haemodynamic consequences, presence and extent of microvascular dysfunction and myocardial fibrosis [4]. The tissue characterisation capabilities of cardiac MRI may help to differentiate HCM from other causes of myocardial thickening and to determine an appropriate treatment strategy [4, 5, 8, 9].

The aim of this article is to illustrate and review the contributions of cardiac MRI to the differential diagnosis among HCM phenotypes and other causes of myocardial thickening.

## Differential diagnosis

There is a broad range of phenotypic expressions of HCM. Asymmetric involvement of the interventricular septum is the most common pattern (60–70%), followed by symmetric or concentric myocardial hypertrophy (up to 40%) and the less common apical variant [4, 8].

From a practical point of view, it is useful to classify the thickening of the myocardium as concentric or symmetric, asymmetric and apical. Table 1 summarises the most common differential diagnosis of HCM phenotypes and the useful cardiac MRI clues suggesting HCM.

**Table 1:** Summary of most common differential diagnosis of HCM phenotypes

Myocardial thickening	Differential diagnosis	Characteristics	MRI clues suggesting HCM
Concentric	Athlete’s heart	Mild wall hypertrophy Increased ventricular volume Normal diastolic function Absence of LGE Detraining can regress the hypertrophy and ventricular volume	Asymmetric wall hypertrophy Small/normal ventricular size Diastolic LV dysfunction LGE can be present
	HHS	Mild wall hypertrophy Elevated indexed LV mass Increased ventricular volume Uncommon mid-wall LGE Regression of LV hypertrophy after systolic blood pressure control	Asymmetric wall hypertrophy Normal indexed LV mass Small/normal ventricular size Patchy LGE most common
	Aortic stenosis	Mild/moderate wall hypertrophy Turbulent flow jet across aortic valve Diffuse subendocardial or mid-wall LGE	Normal aortic root and valve Subaortic turbulent flow jet in obstructive HCM Patchy and extensive LGE most common
	Cardiac amyloidosis	Marked wall thickness Dilatation of both atria Thickening of atrial free wall, interatrial septum and valves Difficult to find the optimal inversion time for nulling the normal myocardium Diffuse, subendocardial or transmural LGE	Asymmetric wall thickness Left atrial dilatation Spared atrial wall, interatrial septum and valves Endocardial LGE is rare
Asymmetric	Cardiac sarcoidosis	Basal septal thinning Aneurysms and ventricular dysfunction Myocardial oedema at T2-w T2 mapping: early detection and follow-up during treatment Basal septal and lateral epicardial LGE	Myocardial oedema at T2-w is uncommon Patchy mid-wall or RV insertion points of ventricular septum LGE
Apical	Mural thrombus	Delayed-enhancement image: very dark thrombus Subendocardial LGE	Delayed-enhancement image: greyish myocardium Patchy mid-wall or RV insertion points of ventricular septum LGE
	LV non-compaction	Apical and mid-wall trabeculations with spared of interventricular septum Non-compacted end-diastolic thickness > 2.3 compacted thickness Cine SSFP images: high signal intensity of intertrabecular recess	Apical myocardial thickening Cine SSFP images: endocardial smooth surface
	Endomyocardial disease	Obliteration of the apical cavity Mural thrombus Subendocardial and triple-layered LGE	Apical myocardial thickening Patchy mid-wall LGE

*LV* left ventricle, *LGE* late gadolinium enhancement, *HHS* hypertensive heart disease, *SSFP* steady-state free precession, *T2-w* T2-weighted imaging

## Concentric LV thickening

HCM with concentric LV hypertrophy should be differentiated from other causes of symmetrical myocardial hypertrophy, including mild (athlete’s heart) and mild or moderate (hypertensive heart disease and aortic stenosis) and from other causes of myocardial thickening (cardiac amyloidosis).

### Athlete’s heart

The term ‘athlete’s heart’ refers to a clinical picture characterised by two distinct and specific cardiac effects induced by a sustained and regular physical training programme, namely, slow heart rate and enlargement of the heart. Increased of LV size and LV hypertrophy are generated in order to normalise LV wall stress. The need for reliable methods to differentiate physiological from pathological LV hypertrophy are brought into focus by the rare but prominent cases of sudden death in elite athletes and the young [10].

**Fig. 1:**
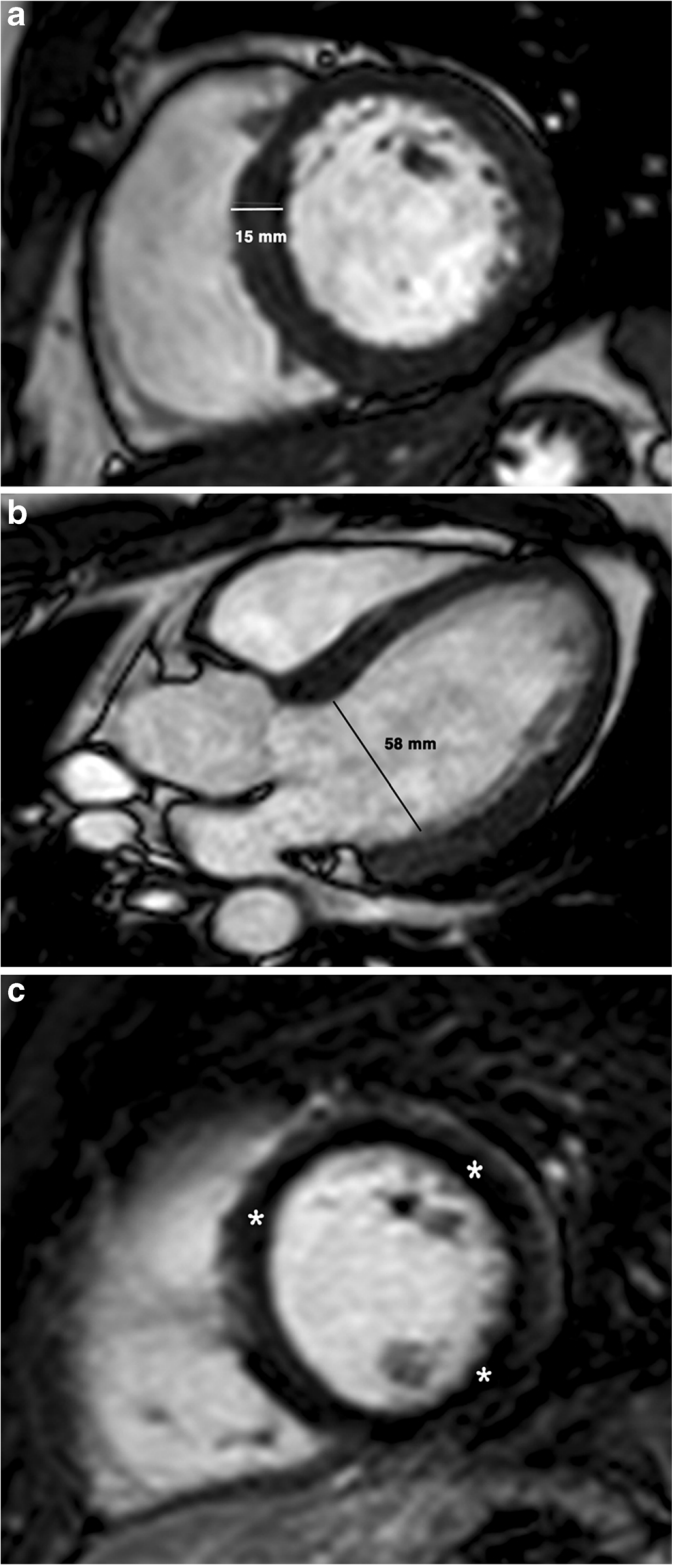
A 20-year-old competitive runner man with athlete’s heart. **a** Short-axis and (**b**) three-chamber steady-state free precession (SSFP) MR images at end-diastole. The left ventricle is enlarged and (end-diastolic diameter = 58 mm) and basal septal thickness is 15 mm. **c** Post-contrast T1-weighted inversion recovery gradient echo image shows normal myocardial signal intensity (*asterisks*)

Cardiac MRI can help to differentiate HCM from the athlete’s heart. In the athlete’s heart, LV wall hypertrophy is concentric, usually mild (≤ 15 mm in male and ≤ 13 mm in female athletes) and is paralleled by a proportional increase in volume of both ventricles (Fig. 1) [10, 11]. Myocardial thickness values greater than 15 mm should be considered definitely abnormal and the diagnosis of HCM should be considered [12]. Patients with athlete’s heart commonly have atrial or ventricular enlargement (LV end-diastolic diameter 55 mm), they respond to temporary discontinuation of exercise training, and they have preservation of the ratio between wall thickness and end-diastolic diameter due to physiological increase in LV volume. Typical values of LV cavity size in athletes with LV hypertrophy range between 55 and 65 mm (Fig. 1b), although up to 10% of athletes with LV hypertrophy exhibit normal LV cavity size [10, 13]. A cut-off for the LV end-diastolic wall thickness (LVEDWT) related to LV end-diastolic volume (LVEDV) of less than 0.15 (LVEDWT/LVEDV < 0.15) [11] and an LV end-diastolic volume related to LV end-diastolic mass (LVEDM) of more than 2.25 (LVEDV/LVEDM > 2.25) in athlete’s heart can help distinguish physiological hypertrophy in athletes from HCM [14].

Patients with HCM have LV hypertrophy with diastolic dysfunction from increased muscle stiffness leading to impaired myocardial relaxation; the ventricular volumes are frequently reduced and the hyperkinetic appearance of systolic contraction translates into a normal or supernormal ejection fraction until the end stage of the disease [1, 4, 15]. Unlike HCM, LV diastolic function is normal in athlete’s heart [10, 11].

Late gadolinium enhancement (LGE) is typically absent (Fig. 1c). Although studies have described small spots of LGE in the septum at the right ventricle (RV) insertion site in athlete’s heart [16, 17], attributed to repetitive myocardial microtrauma, pulmonary artery pressure overload with dilated RV, genetic predisposition and silent myocarditis [17]. On the contrary, the presence of LGE would be suggestive of HCM rather than athletic adaptation [18].

Recent studies have shown that native T1 values and myocardial extracellular volume (ECV) by T1 mapping can be used in the differential diagnosis between HCM and athlete’s heart. While the ECV fraction increases with increasing LV hypertrophy in HCM (due to extracellular matrix expansion and myocardial disarray), the ECV fraction reduces in athletes with an increasing wall thickness (due to an increase in the healthy myocardium by cellular hypertrophy) [19].

The diagnosis of HCM in young competitive athletes may be challenging when the extent of LV hypertrophy is mild and LV wall thickness is in the range of 13–15 mm (12–13 mm in women), which identifies the ‘grey-zone’ of overlap between the physiological adaptations to training and mild phenotypic expression of the disease [10–12]. When the differential diagnosis remains still unresolved, useful information may come from serial echocardiography or cardiac MRI after exercise detraining (3 months) that may show regression of LV hypertrophy and reduction in LV end-diastolic volume in most athletes [10].

- *Practical recommendations: As a general rule, the development of physiological LV hypertrophy in the context of athlete’s heart is consistently associated with an LV cavity—a difference from HCM. When the differential diagnosis remains still unresolved, serial echocardiography or cardiac MRI after exercise detraining (3 months) may show regression of LV hypertrophy and reduction in LV end-diastolic volume in most athletes.*

### Hypertensive heart disease

Arterial hypertension is the most common cause of cardiovascular death. It may lead to hypertensive heart disease and it isthe most common cause of increased afterload that leads to heart failure, ischaemic heart disease and LV hypertrophy [20].

Cardiac MR provides a comprehensive non-invasive evaluation of hypertensive heart disease, including accurate and reproducible assessment of global and regional biventricular function, valvular disease and myocardial fibrosis [21, 22].

In hypertensive heart disease, compensatory LV hypertrophy in response to increased afterload is usually concentric and mild (≤ 13 mm) with an increased indexed LV mass, increased chamber volumes and normal or reduced ejection fraction [5, 21, 23]. Diastolic dysfunction and/or heart failure with preserved ejection fraction due to remodelling of the extracellular matrix and increase in LV filling pressures are common in concentric LV hypertrophy [21, 24]. Myocardial fibrosis plays an important role in the development of diastolic dysfunction. Mid-wall LGE has been documented in patients with hypertensive heart disease, although its prevalence is lower than in HCM patients [21].

The absence of LGE does not equate to the absence of myocardial fibrosis because this LGE identifies focal replacement fibrosis but fails to demonstrate diffuse fibrosis. T1 mapping techniques provide quantification of the myocardial intra and extracellular compartments, and native T1 has demonstrated increased diffuse myocardial interstitial fibrosis at an early stage in hypertensive heart disease patients who do not yet exhibit LGE abnormalities. These abnormalities are associated with decreased LV global function and LV remodelling [25, 26].

**Fig. 2:**
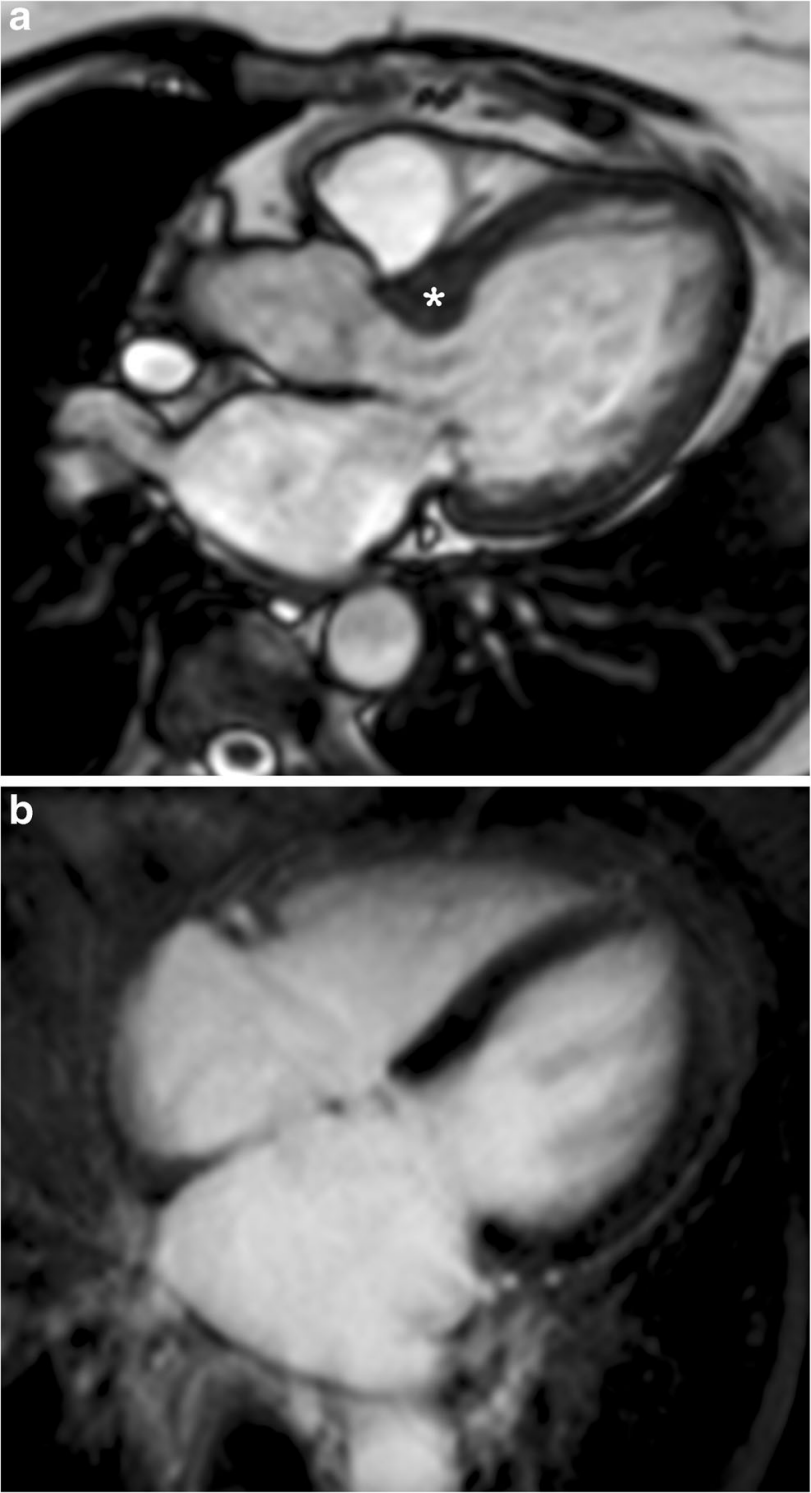
A 64-year-old woman with essential hypertension treated for several years. **a** Three-chamber cine SSFP MR image at end-diastole showing asymmetrical basal septal myocardial hypertrophy (*asterisk*). **b** Four-chamber view of LGE image shows normal dark myocardial signal intensity. Left atrial enlargement also can be seen

When myocardial hypertrophy is ≥15 mm or is asymmetric, distinguishing hypertensive heart disease from HCM can be difficult [23]. Asymmetric basal septal hypertrophy can be seen in up to 10% of cardiac patients without HCM, being more prevalent in the elderly and hypertensives. In these cases, elevated indexed LV mass, increased chamber volumes, normal or reduced ejection fraction and the absence of LGE suggest hypertensive heart disease rather than HCM (Fig. 2). Cardiac MR can also be helpful in detecting changes in serial measurements of LV wall thickness after treatment with antihypertensives, in which regression of hypertrophy supports the diagnosis of hypertensive heart disease [21, 23].

- *Practical recommendations: Independent predictors of hypertensive heart disease rather than HCM are elevated indexed LV mass, absence of myocardial LGE or less pronounced patchy myocardial LGE in hypertensive heart disease than seen in HCM.*

### Aortic stenosis

Aortic stenosis causes a LV pressure overload leading to structural, functional and molecular changes in the process of myocardial hypertrophy. Untreated hypertrophy leads over a longer period of time to ventricular dysfunction that is irreversible and is associated with advanced remodelling [27].

Typically, aortic stenosis presents with mild or moderate concentric hypertrophy because of LV pressure overload with normal LV ejection fraction; however, recent studies have also demonstrated the existence of asymmetrical patterns [28]. Progressive myocardial fibrosis drives the transition from hypertrophy to heart failure in aortic stenosis. Myocardial fibrosis detected by LGE is common and is usually seen in the basal segments, in a diffuse subendocardial or mid-wall distribution. It is irreversible following valve intervention in aortic stenosis and is considered a direct marker of the LV decompensation [29].

**Fig. 3:**
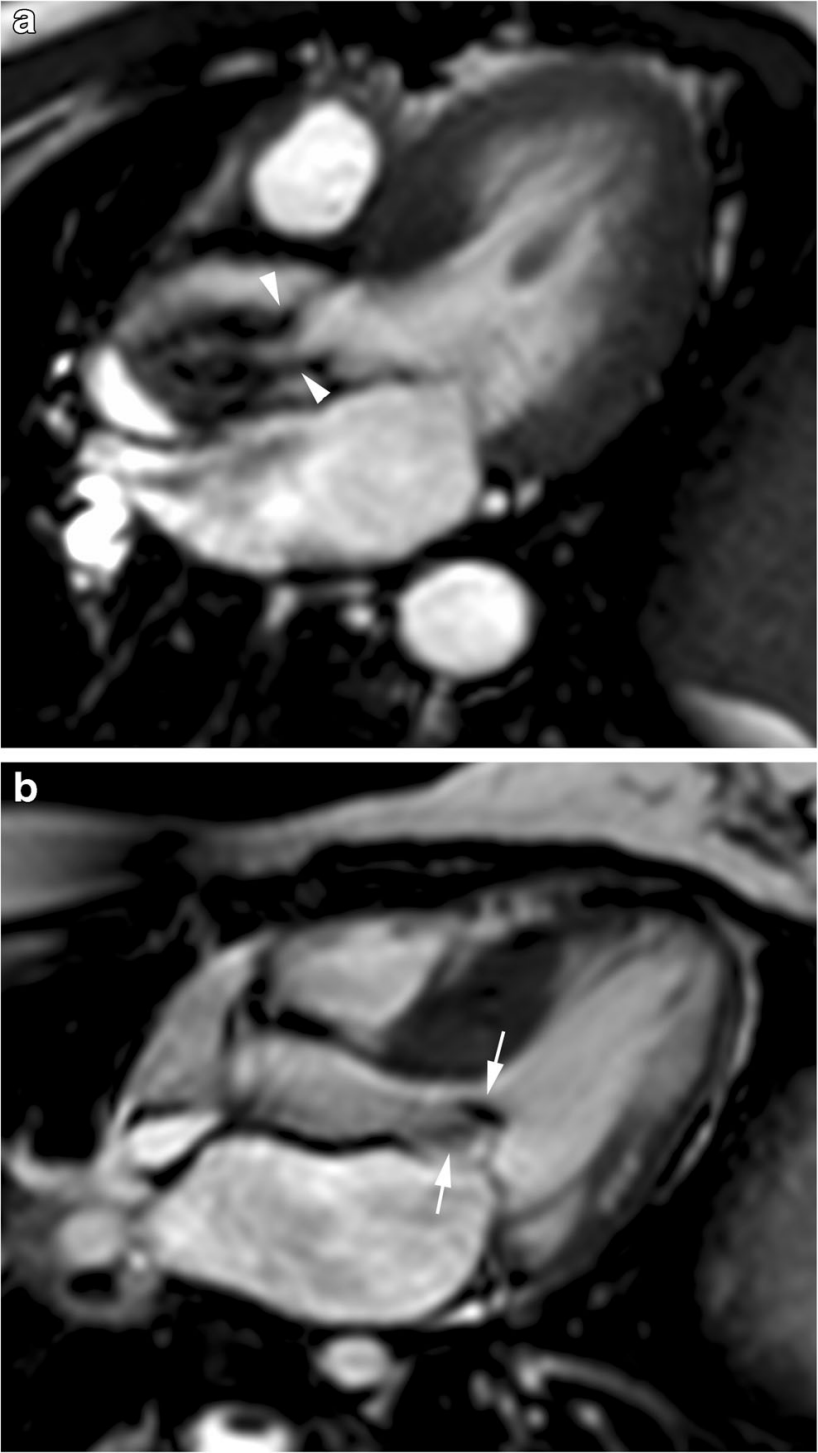
**a** Three-chamber view at end-systole of a 53-year-old man with dyspnea at rest and aortic valve stenosis. **b** Three-chamber view at early systole in a 45-year-old woman with obstructive hypertrophic cardiomyopathy. Jet of turbulent flow is seen exactly across the aortic valve in aortic valve stenosis (*arrowheads*) and in the subaortic region (*arrows*) in obstructive hypertrophic cardiomyopathy with anterior displacement (*arrowhead*) of an elongated anterior mitral leaflet

Cine MR imaging allows differentiation of LV hypertrophy caused by aortic stenosis from that caused by obstructive HCM. In aortic stenosis, the jet of turbulent flow is exactly across the valve with associated decreases in aortic valve area in systole (Fig. 3a). Patients with obstructive HCM have heterogeneous myocardial hypertrophy with thicker basal anterior septal and mid-ventricular inferior septal walls and the jet of turbulent flow is seen in the subaortic region (Fig. 3b) [8, 30].

- *Practical recommendations: Aortic stenosis is readily evaluated on phase-contrast cardiac MRI, and evidence of this finding should be sought when imaging patients for suspected HCM.*

### Cardiac amyloidosis

Cardiac amyloidosis is a rare but important phenocopy of HCM characterised by extracellular deposition of monoclonal light chain or transthyretin amyloid and symptoms of heart failure with preserved ejection fraction. Cardiac involvement in amyloidosis significantly worsens prognosis of the disease. Endomyocardial biopsy is considered the “gold standard” in the diagnosis of cardiac amyloidosis. However, the relatively high risks and clinical complications may hinder its widespread use in clinical settings [31].

**Fig. 4:**
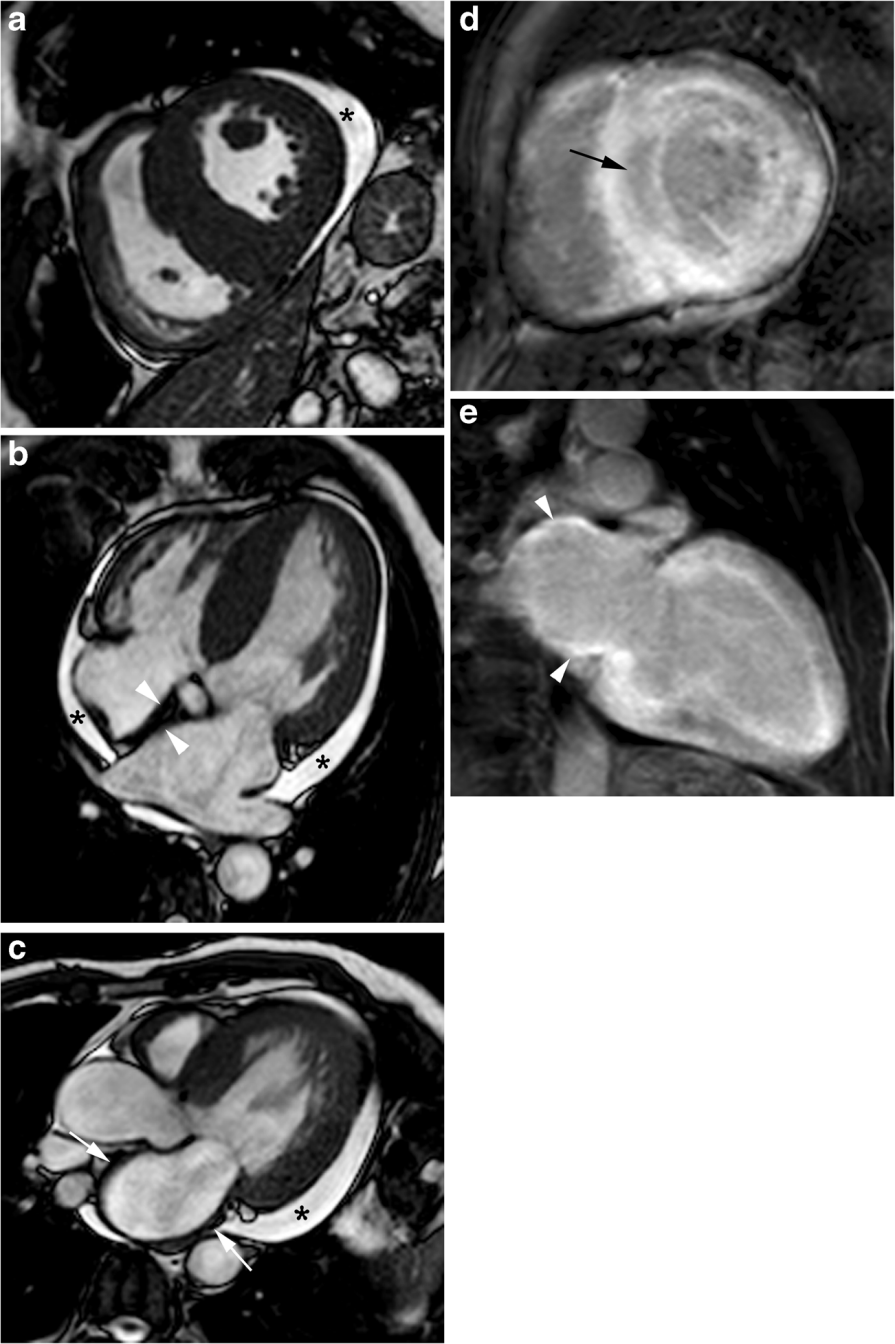
A 65-year-old man with cardiac amyloidosis. **a** Short-axis, (**b**) four-chamber and (**c**) three-chamber cine SSFP MR images at end-diastole show diffuse asymmetric myocardial thickening, of both, LV and RV, thickening of left atrial wall (*arrows*) and interatrial septum (*arrowheads*) and pericardial effusion (*asterisks*). **d** Short-axis and (**e**) two-chamber LGE images showing transmural atrial wall enhancement (*arrowheads*) and diffuse myocardial enhancement sparing the mid-wall of ventricular septum (*arrow*)

**Fig. 5:**
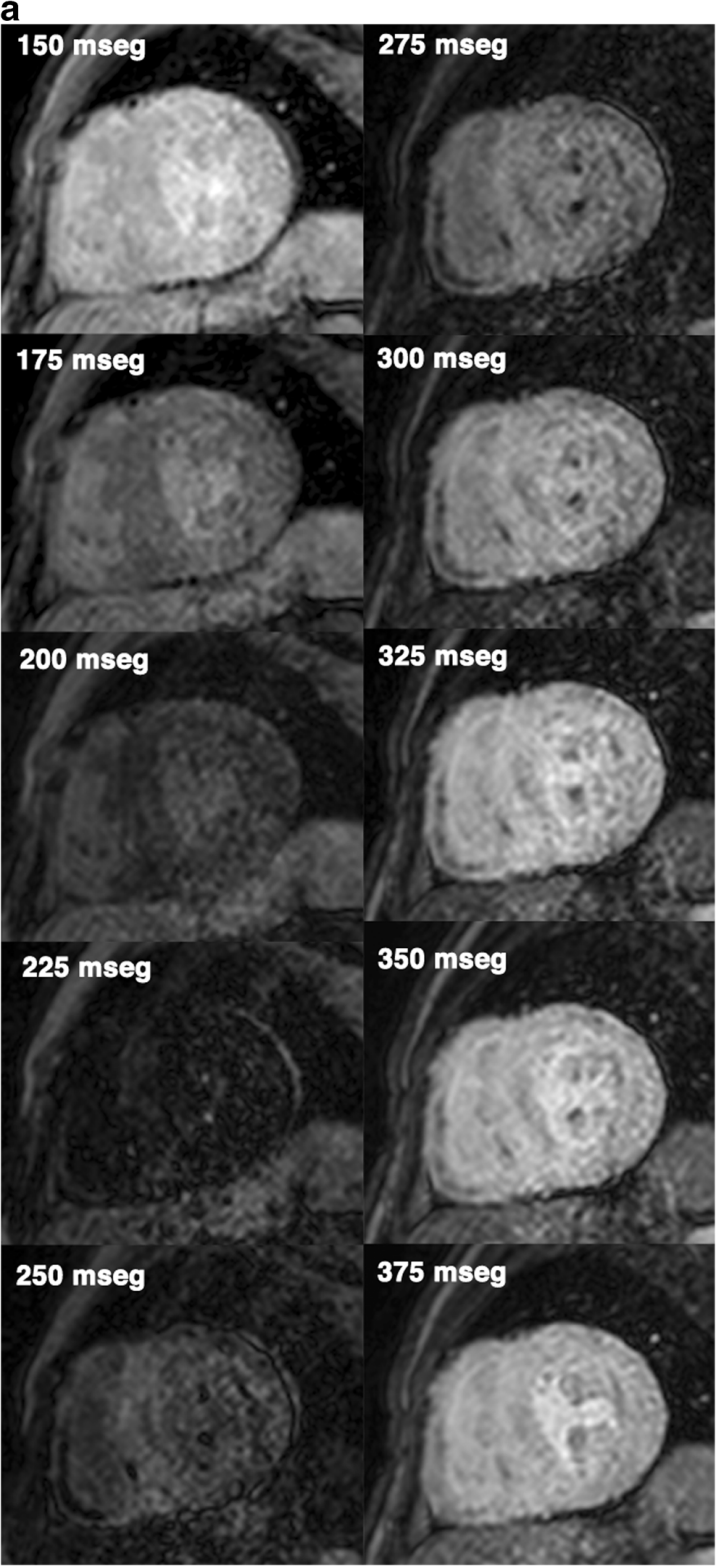

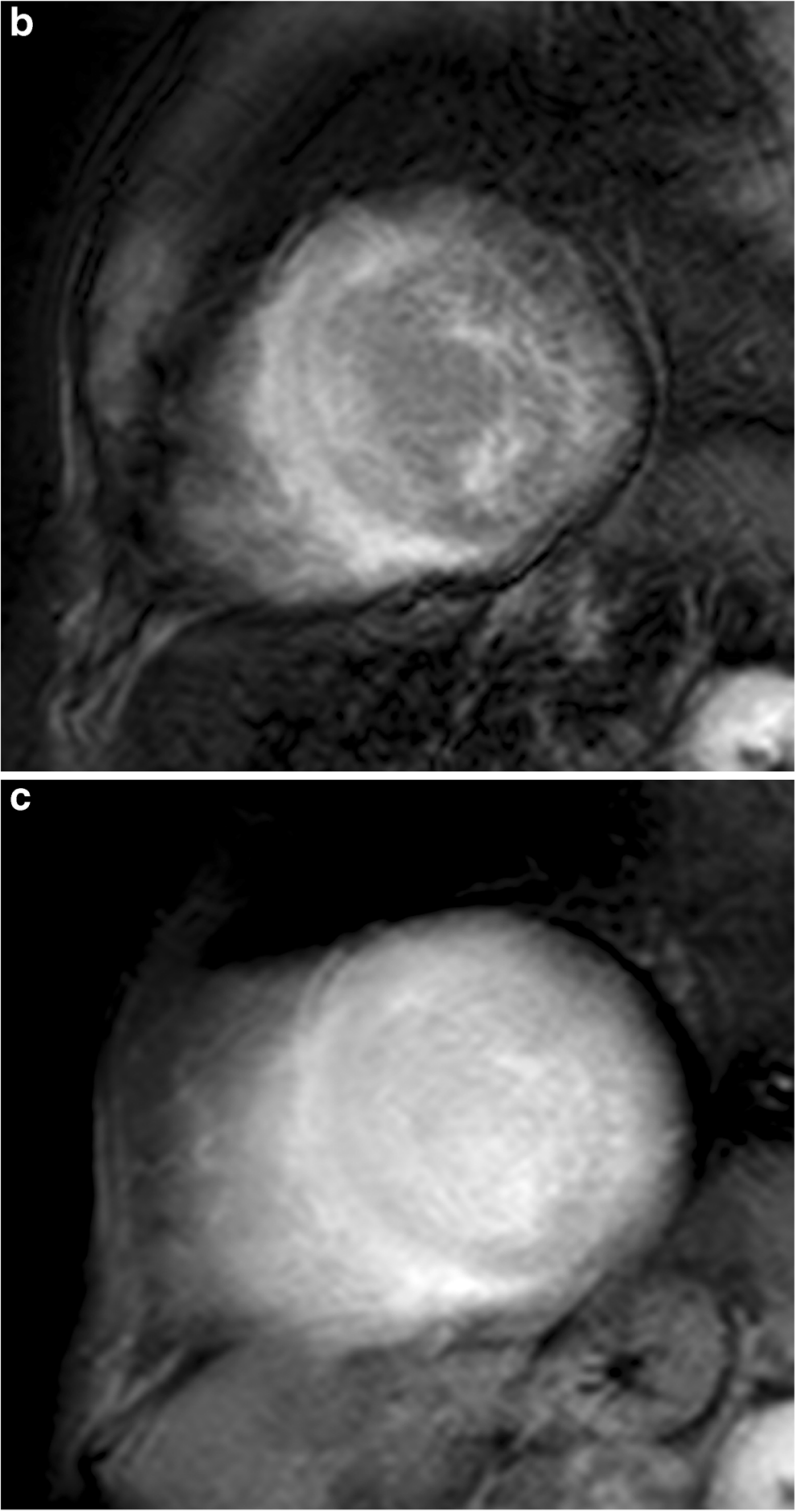
A 75-year-old man with cardiac amyloidosis. **a** Sequential inversion recovery T1-weighted gradient echo images with a variable inversion time trying to null normal myocardium show the difficult to determine the optimal inversion time to null myocardium. The blood pool and the myocardial nulling occurs earlier at 225 ms than the splenic nulling (275 ms). **b** Short-axis T1-weighted inversion recovery gradient echo images obtained at (**b**) 4 minutes and (**c**) 8 minutes after the injection of contrast agent. The myocardium displays predominantly subendocardial LGE at 4 minutes, but it is diffusely enhanced at 8 minutes

Cardiac amyloidosis most commonly presents with markedly symmetric LV thickening (Figs. 4 and 5), dilatation of both atria, decreased LV volumes, diastolic dysfunction with restrictive pattern and pericardial and pleural effusions [32, 33]. Asymmetric obstructive LV thickening has also been described, mimicking HCM [32, 34]. Morphological changes of a thickened right atrial free wall, interatrial septum and valves are helpful in distinguishing cardiac amyloid from HCM (Fig. 4b and c) [32].

Cardiac MRI with LGE provides unique information regarding myocardial tissue characterisation and it is extremely helpful in differentiating cardiac amyloidosis from HCM. Due to interstitial expansion from amyloid deposition, LGE is seen in 69–97% of all cardiac amyloidosis patients [5, 32].

Alterations in gadolinium kinetics in the blood and myocardium are common and can be useful in differentiating cardiac amyloidosis from HCM [35]. The high tissue uptake and faster washout of gadolinium from blood and myocardium may result in perceived difficulties in selecting an appropriate inversion time to null the myocardial signal on the delayed enhancement imaging pulse sequence [32, 35]. At 4 min after gadolinium administration, the inversion time of myocardium affected by amyloid is shorter than normal and lengthened with time. T1 values of subendocardium and subepicardium are similar between 8 and 10 min after gadolinium administration owing to altered contrast agent kinetics and therefore imaging must be performed earlier than usual and completed quickly [35] (Fig. 5).

In cardiac amyloid disease, the deposition of the abnormal protein typically occurs in a circumferential manner starting in the endocardium and then extending to the myocardium in a transmural fashion. Characteristic patterns of myocardial enhancement include global, subendocardial and, less often, patchy or diffuse LGE distribution within the LV (Fig. 4d, e) [32, 33, 36, 37]. Recently, lower LGE in the apical myocardial segments compared to the basal segments has been reported [38].

Higher ECV post-contrast T1 mapping correlates with LGE, indexed LV mass and other clinical adverse prognostic factors [5]. In early disease, native T1 and equilibrium contrast enhancement MR imaging are elevated before LGE appears and correlate with the severity of cardiac amyloid deposition and with markers of systolic and diastolic dysfunction [36, 37].

- *Practical recommendations: Late imaging with inversion recovery should be performed at 4 min and completed quickly to identify myocardial amyloid deposition.*

#### Asymmetric LV thickening

Asymmetric LV hypertrophy is the most common phenotypic expression of HCM, which typically involves the basal ventricular septum. Infiltrative cardiomyopathies usually cause symmetrical LV wall thickening; however, occasionally cardiac sarcoidosis may manifest as asymmetric LV wall thickening simulating HCM.

### Cardiac sarcoidosis

Sarcoidosis is a multisystem disorder of unknown aetiology that is characterised histopathologically by non-caseating granulomatous infiltration. Cardiac involvement is common (50%), but only 5% of patients are symptomatic and may initially manifest with arrhythmias or even sudden cardiac death [39].

Disease may involve either the left or right ventricle but more commonly involves the LV, usually the basal septum; nevertheless, involvement of RV free wall, atrium, pericardium and endocardium can also be seen [39, 40].

Abnormalities of cardiac sarcoidosis tend to be non-specific and variable; interventricular thinning (particularly basal) is the most typical feature of cardiac sarcoidosis [39]. There may be other abnormalities, such as aneurysms, LV and/or RV diastolic and systolic dysfunction, regional wall motion abnormalities, LGE and myocardial oedema [39, 40].

The appearance of sarcoidosis at cardiac MRI largely depends on the timing of imaging. In the acute phase of disease, myocardial inflammation or oedema manifests as myocardial thickening and patchy increased signal intensity on T2-weighted images and T2 mapping. Recent studies have shown that T2 mapping seems to serve as a novel quantitative biomarker to detect myocardial inflammation in systemic sarcoidosis and during the follow-up of the disease. T2 values are higher in cardiac sarcoidosis than in patients without cardiac involvement and decrease in response to anti-inflammatory treatment. [41].

LGE MR images in patients with sarcoidosis typically show a patchy mid-myocardial, subepicardial or epicardial pattern that is not in a vascular distribution, most often seen in basal segments (particularly of the septum and lateral wall) and typically in the epicardium and mid myocardium [39]. In chronic disease, nodular foci of LGE indicative of fibrosis and scar formation without corresponding T2-weighted signal intensity may be present [39, 41, 42].

**Fig. 6:**
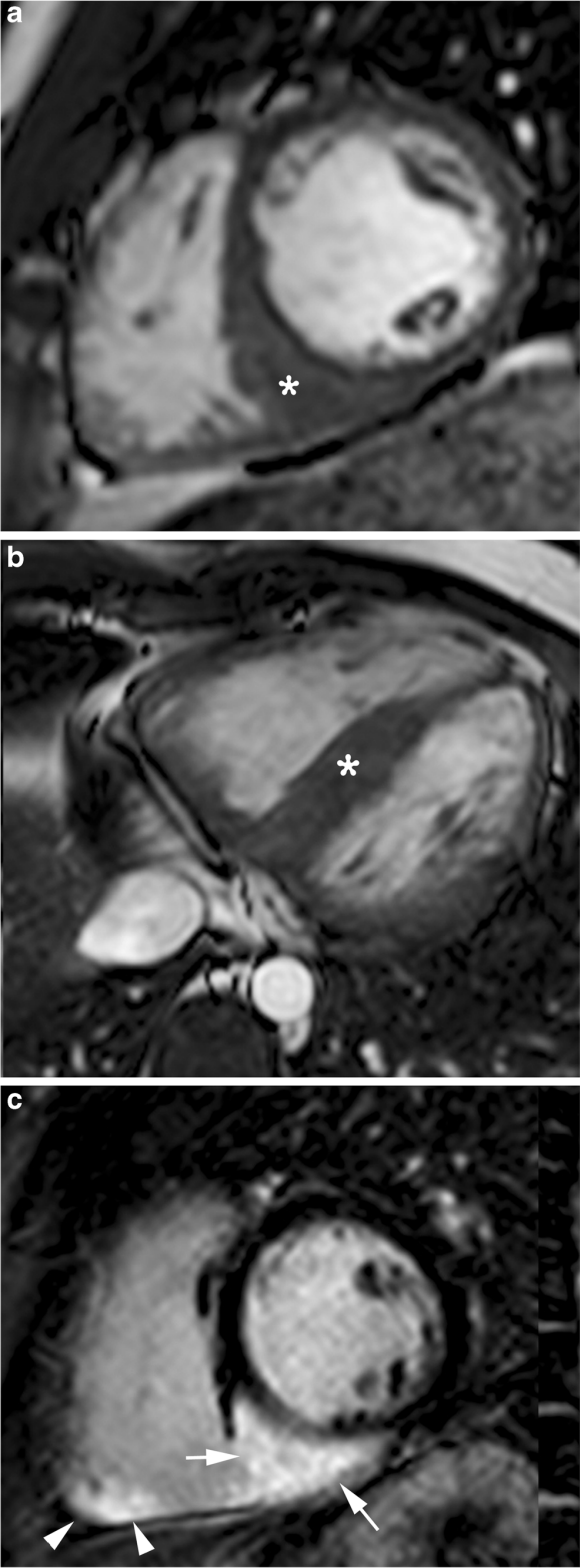
A 29-year-old man with pulmonary sarcoidosis who had recurrent ventricular tachycardia and negative coronary angiography findings. **a** Short-axis and (**b**) axial cine SSFP MR images at end-diastole show basal septal myocardial thickening (*asterisks*). **c** Short-axis post contrast T1-weighted inversion recovery gradient echo image shows subepicardial LGE (*arrows*) in the inferoseptal and inferior walls of the left ventricle, as well as subendocardial LGE (*arrowheads*) in the inferior wall of the right ventricle. Functional assessment (not shown) demonstrated 35% left ventricular ejection fraction

Diagnosis of cardiac sarcoidosis is sometimes challenging because sarcoidosis often involves small areas of the myocardium without abnormally affecting LV function and, less commonly, an increase in myocardial wall thickness may also be seen, usually at the basal septum, simulating asymmetric HCM (Fig. 6a and b) [43, 44]. In these cases, T2-weighted MR imaging and LGE are useful to suggest the diagnosis. Unlike cardiac sarcoidosis, which is characterised by inflammation, HCM is not commonly seen with oedema on T2-weighted MR imaging. Furthermore, LGE in cardiac sarcoidosis is more likely to be epicardial (Fig. 6c), while in HCM, LGE usually involves the anterior and posterior junctions of the RV free wall and interventricular septum [39].

- *Practical recommendations: T2 mapping can be useful for early detection of cardiac involvement in systemic sarcoidosis and for monitoring the activity of myocardial inflammation during the follow-up of the disease.*

## Apical LV thickening

The differential diagnosis of apical HCM includes mural thrombus, hypertrabeculation or non-compaction and endomyocardial fibrosis. These entities may be diagnosed on MRI using steady-state free precession (SSFP) imaging techniques and LGE imaging.

### Mural thrombus

The development of LV apical thrombus is an important complication of myocardial infarction. Thrombus occurs on the endocardial surfaces overlying the infarct secondary to endocardial inflammation during the acute phase of myocardial infarct. Although the underlying pathological mechanisms are unknown, approximately 2% of patients with mid-ventricular obstruction in HCM present with apical LV aneurysms. The dyskinetic/akinetic apex can provide the structural basis for intracavitary thrombus formation [45]. Echocardiographic distinction between LV apical thrombus and apical HCM can be difficult. In these cases, cardiac MRI using delayed-enhancement image with a long inversion time (500–600 ms) can differentiate mural thrombus from myocardial hypertrophy and other cardiac masses because the blood pool and myocardium tend to become greyish while the thrombus remains dark [46].

- *Practical recommendations: Late imaging with inversion recovery should be performed with a long inversion time (500–600 ms). At this time, the signals of thrombi are very dark.*

### LV non-compaction

**Fig. 7:**
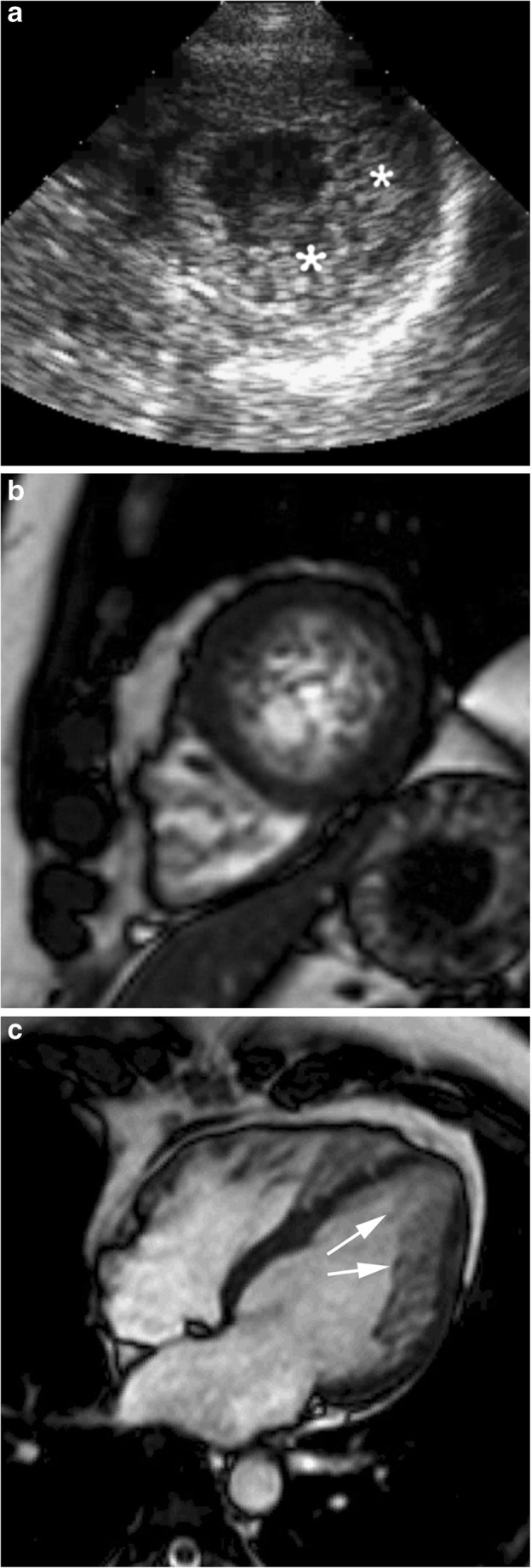
A 45-year-old woman with reduced ejection fraction and isolated LV non-compaction. **a** Echocardiographic findings were suggestive of apical hypertrophic cardiomyopathy (*asterisks*). **b** Short-axis cine SSFP MR image at end-diastole shows left ventricular apical trabeculated myocardium and thin epicardial compacted layer. End-diastolic ratio between non-compacted and compacted layers is greater than 2.3. **c** End-diastolic long-axis view SSFP image demonstrates the myocardial trabeculations (*arrows*) and clearly depicts their extension from apex to lateral wall

Left ventricular non-compaction is a genetic cardiomyopathy characterised by an excessively prominent trabecular meshwork and deep intertrabecular recesses that communicate with the cavity but not with the coronary artery system [47]. Non-compacted areas are commonly located at the LV apex and mid-apical wall segments, typically sparing the interventricular septum (Fig. 7) [47–49].

Patients may have no symptoms or may present with heart failure, atrial and ventricular arrhythmias, thromboembolic events and sudden cardiac death [50].

Although a “gold standard” for the diagnosis of LV non-compaction continues to be lacking, cardiac imaging criteria provide the best tool currently available [47]. Imaging diagnostic criteria are based on the relationship between the thicknesses of the non-compacted and compacted layers. An end-systolic ratio between non-compacted and compacted layers greater than 2 in the short-axis view is considered diagnostic on echocardiography [48].

Advances in cardiac MRI have resulted in superior image quality and increased sensitivity in the detection of myocardial trabeculations. Moreover, cardiac MR can also reveal the presence of LGE, a marker of myocardial fibrosis that represents the substrate for potentially lethal arrhythmias [51]. Higher prevalence of LGE is associated with disease severity and LV systolic dysfunction [47].

On cardiac MRI, diagnosis of LV non-compaction is supported if the end-diastolic thickness of the non-compacted layer is greater than 2.3 times that of the compacted one [52]. This relationship should be measured in short-axis images when compacted and non-compacted myocardium is located in the mid-cavity and basal segments. When myocardial trabeculations are located at the apex, the four-chamber or long-axis views are preferred [47, 49].

The diagnosis of LV non-compaction can be challenging due to the lack of universally validated diagnostic criteria [47, 52]. Diagnosis is also complicated by the fact that there is a complex genetic background responsible for isolated LV non-compaction development that is in part shared with hypertrophic and dilative cardiomyopathy [47, 53].

Left ventricular non-compaction shares morphological features with HCM that can mimic isolated LV non-compaction [48]. A true overlap may exist, as reported in genotyped families expressing both HCM and LV non-compaction phenotypes, and both diseases can occur in the same patient [47, 54].

Trabeculations of LV non-compaction at echocardiography can simulate an apical HCM because myocardial trabeculations can be difficult to visualise in the apical segments (Fig. 7a) [47, 55]. The high signal intensity of the blood pool achieved by cine SSFP MR images allow reliable differentiation of compacted and non-compacted layers of the LV myocardium corresponding to areas characterised by myocardial thickening on echocardiography (Fig. 7b and c). Unlike LV non-compaction, cine SSFP MR images easily demonstrate the apical myocardial thickening and the smooth surface of the endocardium without trabeculations characteristic of apical HCM [56].

- *Practical recommendations: The intertrabecular recesses that communicate with the LV cavity and that are characteristic of LV non-compaction are easily demonstrated by the high signal intensity of the blood pool on cine SSFP MR images.*

### Endomyocardial disease

**Fig. 8:**
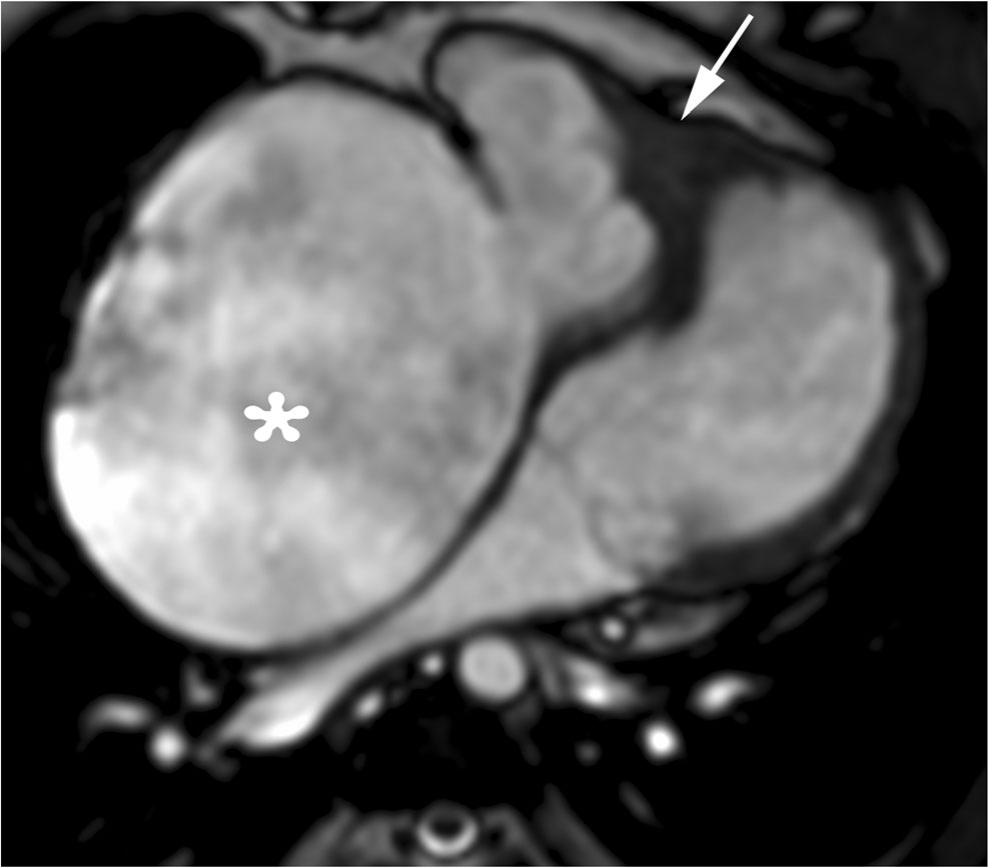
A 22-year-old woman with endomyocardial fibrosis. End-diastolic four-chamber cine SSFP MR image shows right ventricular apical thickening (*arrow*) and marked right atrial dilatation (*asterisk*) with signal void attributed to turbulent blood flow

**Fig. 9:**
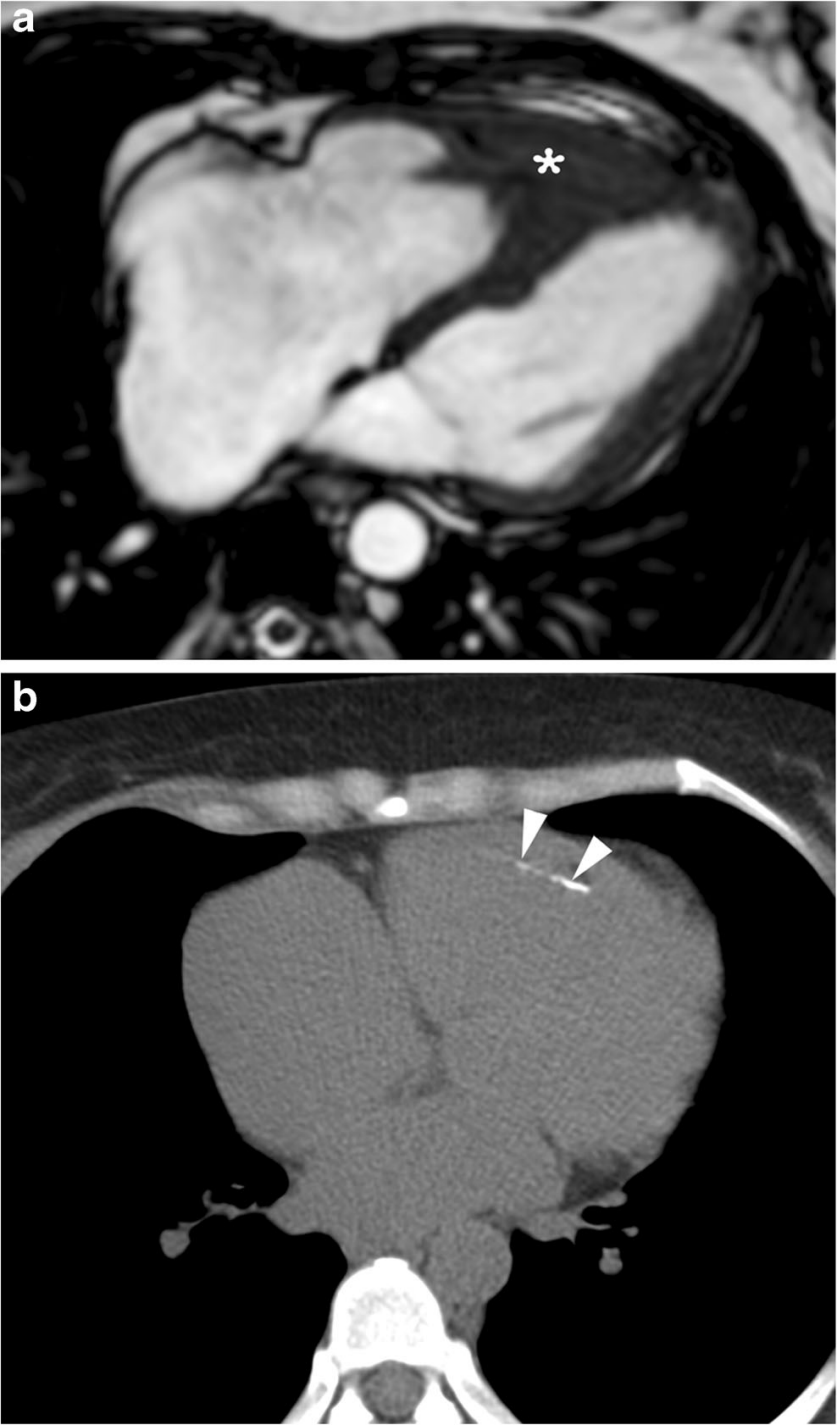
A 39-year-old woman with endomyocardial fibrosis. **a** Four-chamber view cine SSFP MR image at end-diastole shows right atrial dilatation and myocardial thickening (*asterisk*) with obliteration of the right ventricular apical chamber. **b** Non-enhanced thoracic CT scan showing endocardial calcification (*arrowheads*)

Hypereosinophilic syndrome with cardiac involvement and endomyocardial fibrosis is the most prevalent form of restrictive cardiomyopathy worldwide. This entity usually involves the apex of one or both ventricles that may lead to both endomyocardial fibrosis and obliteration of the LV apical cavity (Figs. 8 and 9) [57].

Fibrosis at the cardiac apex frequently results in obliteration of the apical cavity, and can be confused with apical HCM [58]. On cardiac MRI, a high T2 signal may be seen in the apical endocardium. Diffuse subendocardial perfusion defects and distortion of the mitral valve apparatus with resultant regurgitation may be present [59, 60]. Superimposed mural thrombus and endocardial calcification may also be seen in advanced cases (Fig. 9b) [59, 61]. Subendocardial LGE is common and a triple-layered pattern of enhancement is a characteristic. This includes an inner dark layer due to non-enhancing thrombus, a middle bright layer due to LGE from fibrous tissue and an outer dark layer of normally nulled myocardium [5, 59]. Patchy intramyocardial LGE and associated wall motion abnormalities and dilated left atrium may also be seen [5, 59, 60].

- *Practical recommendations: Mural thrombus and subendocardial LGE in endomyocardial fibrosis are most useful findings to differentiate this entity from apical HCM.*

## Conclusions

In summary, familiarity with the spectrum of myocardial thickening mimickers allows consideration of the differential diagnosis of HCM. Understanding relevant clinical features, the myocardial thickening location and distribution patterns of late gadolinium enhancement facilitates the recognition of key cardiac MRI features, which can allow identification of those causes of myocardial thickening that may mimic the various HCM phenotypes.
